# Factors Associated With False-Negative Endoscopic Biopsy Results After Neoadjuvant Chemoradiotherapy in Patients With Esophageal Squamous Cell Carcinoma

**DOI:** 10.1097/MD.0000000000000588

**Published:** 2015-02-27

**Authors:** Yin-Kai Chao, Chi-Ju Yeh, Mu-Hsien Lee, Yu-Wen Wen, Hsien-Kun Chang, Chen-Kan Tseng, Yun-Hen Liu

**Affiliations:** From the Division of Thoracic and Cardiovascular Surgery (Y-KC, Y-HL); Department of Pathology (C-JY); Division of Gastroenterology (M-HL); Clinical Informatics and Medical Statistics Research Center (Y-WW); Division of Hematology/Oncology (H-KC); and Department of Radiation Oncology (C-KT), Chang Gung Memorial Hospital, Linkou, College of Medicine, Chang Gung University, Taoyuan, Taiwan.

## Abstract

The usefulness of endoscopic biopsy following neoadjuvant chemoradiotherapy (nCRT) is limited because of its high false-negative (FN) rates. However, data on the factors associated with FN biopsy results remain scarce. The purpose of this study was to investigate factors associated with FN results on endoscopic biopsies in patients with esophageal squamous cell carcinoma (ESCC) following nCRT.

We retrospectively reviewed the records of ESCC patients who were treated at the Chang Gung Memorial Hospital, Taoyuan, Taiwan, between 1999 and 2013. Inclusion criteria were receiving nCRT as first-line treatment before esophagectomy and having been preoperatively submitted to an endoscopic biopsy. Endoscopic findings at the lesion site were classified into 6 distinct categories: stricture, tumor, ulcer, scar, other findings, or normal. Univariate and multivariate analyses were used to identify factors associated with FN biopsy findings.

A total of 227 patients were selected, of which 92 (41.9%) had positive biopsy results. Among patients with negative biopsy findings (n = 135), 85 were found to have residual cancer on the resected esophagus. Multivariate analysis identified endoscopic findings as the only independent predictor of FN biopsy results. The negative predictive values were 77.8%, 61.9%, 52.6%, 30.3%, 23.1%, and 20.0% for the normal, scar, other findings, ulcer, stricture, and tumor categories, respectively (*P* < 0.001).

In ESCC patients, the FN rate of endoscopic biopsy after nCRT is associated with the type of residual lesion.

## INTRODUCTION

Neoadjuvant chemoradiotherapy (nCRT) is increasingly being used as a first-line treatment modality in patients with esophageal cancer.^1^ Despite the use of various chemotherapy regimens and radiation doses, at least 20% to 40% of patients treated with nCRT are expected to achieve a pathological complete response (pCR), defined as the absence of viable cancer cells in the resected esophagectomy specimens.^2–4^ Although esophagectomy does not confer an obvious benefit to patients who achieve pCR, the preoperative identification of such patients remains difficult. Endoscopic biopsy is one of the most common diagnostic modalities used to identify pCR following nCRT. However, its accuracy for identifying the presence of residual cancer cells following nCRT remains problematic. It has a high likelihood of missing the presence of an actual cancer and is considered too inaccurate for treatment decision guidance; the reported negative predictive value (NPV) is 11% to 36%.^5–9^ In this context, the decision to avoid surgery based on complete reliance on negative biopsy findings may ultimately result in local recurrences in the majority of patients. Because of this, several institutions do not routinely perform endoscopic biopsies following nCRT. Alternative approaches, including endoscopic ultrasound and positron emission tomography (PET), have been utilized for identifying patients who achieved pCR, but the results remain suboptimal.^9–13^

Theoretically, the power of a diagnostic test is significantly influenced by the prevalence of the disease. Specifically, the positive predictive values (PPVs) and NPVs can change dramatically depending on the disease prevalence.^14^ In this regard, an increase in pCR rates (ie, the prevalence of nondisease) in patients with esophageal cancer treated with nCRT ultimately results in a corresponding increase in NPV.^14^ Esophageal squamous cell cancer (ESCC) is known to be more sensitive to chemoradiation than adenocarcinoma and has higher pCR rates following nCRT.^15^ Furthermore, recent advances in radiotherapy techniques and chemotherapy regimens have significantly increased the efficacy of nCRT, and pCR rates are continuously rising. A recent randomized trial reported pCR as high as 49% in the ESCC subgroup.^16^ Based on these encouraging results, we reasoned that the use of endoscopic biopsies might feasibly identify pCR in ESCC patients. Unfortunately, the extant evidence in support of our hypothesis is scarce and mostly obtained in small-sized studies focusing on a limited number of ESCC patients. We therefore designed this study to investigate the clinical value of endoscopic biopsies in ESCC patients following nCRT. In contrast to previous studies, we classified endoscopic findings after nCRT into 6 distinct categories: normal, scar, ulcer, other findings, stricture, or tumor. Univariate and multivariate analyses were also used to identify the main factors associated with false-negative (FN) biopsy findings.

## MATERIALS AND METHODS

### Patients

We retrospectively reviewed the records of consecutive ESCC patients who were referred to the Chang Gung Memorial Hospital, Taoyuan, Taiwan, between 1999 and 2013. Inclusion criteria were presence of locally advanced disease requiring nCRT as first-line treatment before esophagectomy and having been submitted to an endoscopic biopsy following nCRT.

Pretreatment staging was based on the results of computed tomography (CT) of the chest and abdomen, esophagography, endoscopic ultrasound, and PET (for patients treated after 2005). Pretreatment tumor length was defined as the maximum tumor length measured using a barium contrast agent. Staging was performed according to the American Joint Committee on Cancer Staging Manual, Seventh Edition. The exemption from retrospective review and data collection methods was made by the Institutional Review Board, Chang Gung Memorial Hospital. The date of the last follow-up was May 31, 2014.

### nCRT and Restaging Workup

The preoperative chemoradiotherapy regimens were as follows: 5-fluorouracil (1000 mg/m^2^/d) was administered as a continuous infusion over 96 hours from days 1 to 4 and from days 29 to 33, and cisplatin (75 mg/m^2^) was administered as an intravenous infusion over 3 hours on days 1 and 29. Radiation therapy between days 8 and 29 consisted of a total dose of 30 Gy, administered in daily fractions of 200 cGy, 5 days per week. A complete restaging workup that included chest-to-abdomen CT, endoscopy, esophagography, and PET scan was performed 4 to 6 weeks following the completion of nCRT.

### Evaluation of Clinical Response by Endoscopy

**FIGURE 1:**
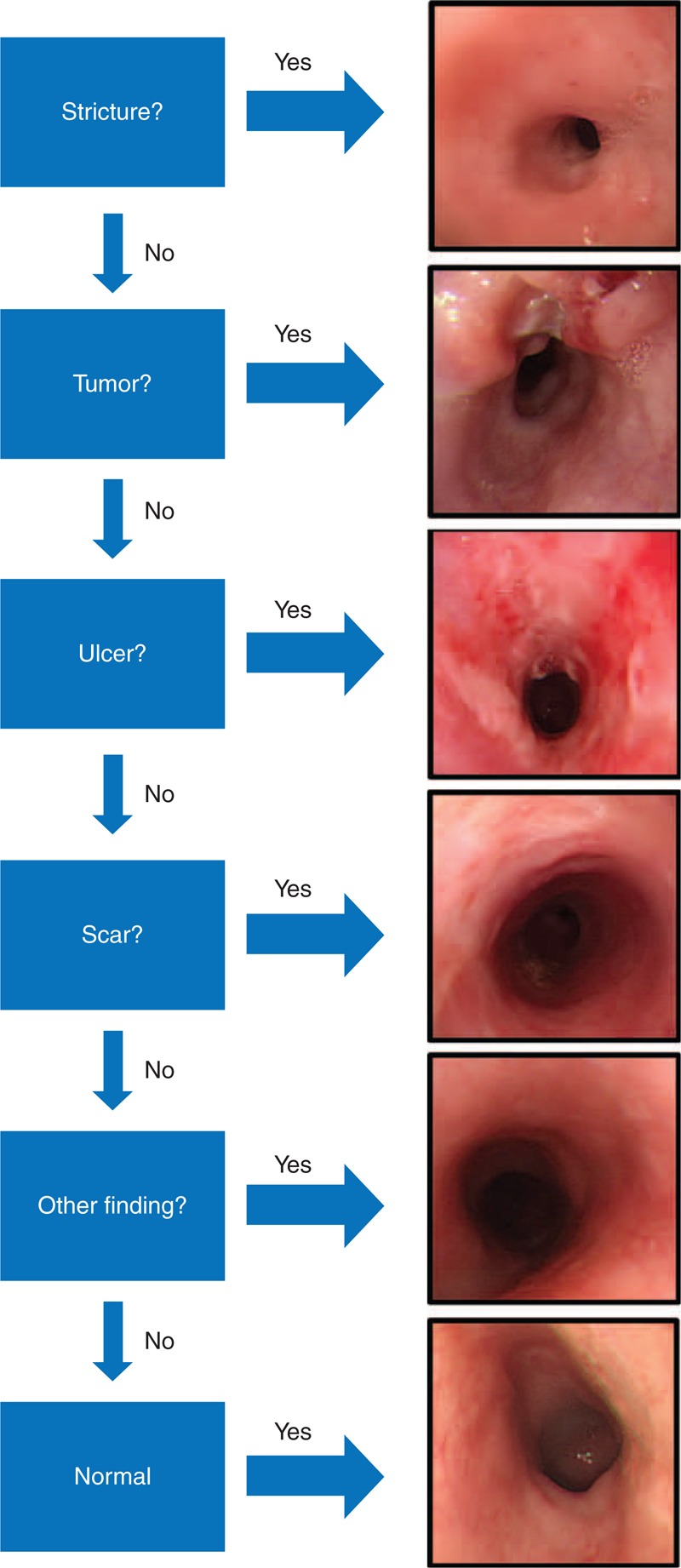
Decision process during endoscopic examination and representative picture of each category.

Restaging endoscopic evaluations were performed by 3 experienced gastroenterologists (Yin-Yi Chu, Mu-Hsien Lee, and Cheng-Tung Chiu, Division of Gastroenterology, Chang Gung Memorial Hospital, Linkou, College of Medicine, Chang Gung University, Taoyuan, Taiwan) who were blinded to the results of all other staging procedures. Classification of the endoscopic findings into the 6 categories is summarized in Figure 1. Briefly, a standard endoscope (9.8 mm in diameter) was used to evaluate the esophagus, and a classification of “stricture” was assigned when the endoscope failed to pass through the site of the original esophageal lesion.^17^ If no stricture was present, a through endoscopic examination was performed to determine classifications of “tumor,” if any residual tumor was found, of “ulcer,” if an active ulcer was found, or of “scar,” if the ulcer was healed. The remaining abnormal mucosal findings (eg, mucosa tag, polypoid lesion, granular protruded lesions, erosion, and lugol-voiding lesions) were classified as “other findings.” Finally, patients who did not show any mucosal abnormality were classified as “normal.”

### Surgical Resection Following nCRT and Postoperative Adjuvant Therapy

In the absence of contraindications, esophagectomy was scheduled in all patients. Eligibility for surgery was based on the following: medical fitness for surgery, with absence of liver cirrhosis and/or heart failure (New York Heart Association class III or IV); absence of tracheoesophageal fistula; and no evidence of recurrent laryngeal nerve invasion.

The standard surgical approach consisted of a limited thoracotomy on the right side followed by an intrathoracic gastric tube reconstruction (Ivor-Lewis procedure) for lesions located in the middle and lower one-third of the esophagus. Lesions of the upper one-third of the esophagus or cervical lesions were treated by neck anastomosis (McKeown procedure). All patients underwent a 2-field lymph node dissection. Pyloroplasty and feeding jejunostomy were not routinely performed. A nasogastric tube was placed in each patient until the anastomotic sites were closed, based on the results of esophagography performed on postoperative day 14.

### Pathological Examination

Slices obtained from endoscopic biopsies or esophagectomy were stained with hematoxylin and eosin and microscopically examined to confirm the presence or absence of cancer cells. Specimens obtained from esophagectomy were opened longitudinally and fixed in 10% formaldehyde overnight. In the presence of residual tumors, representative sections were carefully examined for determining the maximal depth of invasion and the reciprocal relationships with both the esophagus and the stomach. In the absence of gross tumors, ulcerated or fibrotic areas were sampled and representative sections were submitted for examination. We defined complete pCR as the absence of tumor cells in all the operative pathological specimens, including both the primary site and the sampled lymph nodes. Local pCR was defined as the absence of cancer cells in the esophagus, regardless of the nodal status.

### Posttherapy Surveillance

The study subjects were scheduled for chest x-rays every 3 months and for CT every 6 months postoperatively. Patients with recurrent symptoms underwent panendoscopy. Survival was assessed every 6 months through contact with the patient's physicians or a review of medical records. In case of missing information, data were retrieved from the National Cancer Registry Database of Taiwan.

### Classification of Endoscopic Biopsies

Biopsies were considered as FN if they yielded negative findings in patients showing evidence of a residual tumor at the primary site (ypTx). We calculated the sensitivity, specificity, PPV, NPV, and accuracy based on the results of histology examinations using either the surgical specimens or endoscopic biopsies. We defined sensitivity as the number of true-positive cases divided by the sum of true-positive and FN cases. Specificity was calculated as the number of true-negative cases divided by the sum of true-negative and false-positive cases. We determined the PPV by dividing the number of true-positive cases by the sum of true-positive and false-positive cases. The NPV was calculated by dividing the number of true-negative cases by the sum of true-negative and FN cases. Diagnostic accuracy was defined as the sum of true-positive and true-negative cases divided by the total number of cases.

### Data Analysis

The SPSS statistical software, version 12.0 (SPSS Inc, Chicago, IL), was used for all analyses. Continuous variables were given as means ± standard deviations and differences were analyzed using the Student *t* test. Categorical data were presented by frequency counts, and intergroup comparisons were performed using the χ^2^ test. Variables with univariate *P* values <0.15 were entered as covariates into a multivariable regression model. Results for the multivariable regression analysis were expressed as odds ratios with their 95% confidence intervals (CIs). A probability value *P* < 0.05 (2-tailed) was considered statistically significant.

## RESULTS

### General Characteristics of the Study Participants

**FIGURE 2:**
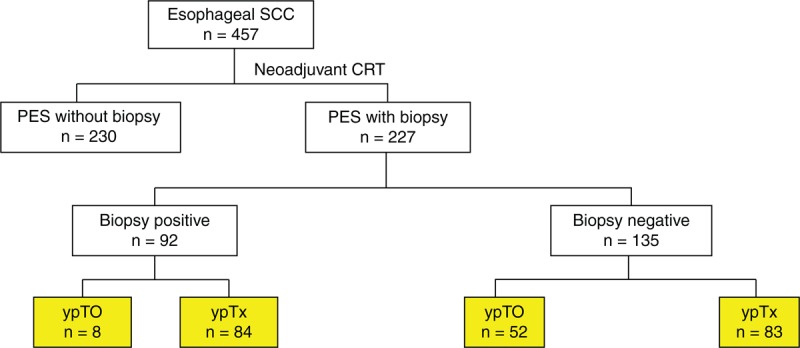
Flow diagram of patient selection. CRT = chemoradiotherapy, PES = panendoscopy, SCC, squamous cell carcinoma.

A flow diagram of patient selection is shown in Figure 2. Between January 1999 and October 2013, we identified a total of 457 ESCC patients who underwent nCRT followed by surgery. Of those, 227 underwent endoscopic biopsy before surgery. The decision to perform this biopsy was made by the treating oncologists based on clinical judgment. General characteristics of the entire cohort are summarized in Table 1. There were 222 males and 5 females, with a mean age of 55.6 years (range, 31–78 years). Most tumors occurred in the middle-third of the esophagus (59%, 134/227). According to the results of esophagography, the mean pretreatment tumor length was 6.1 cm (range, 1.5–16 cm). Sixty (26.7%) of the 227 patients achieved local pCR (ypT0). Among them, 54 had complete pCR (ypT0N0). The Ivor-Lewis procedure was used in 168 individuals, whereas the McKeown procedure was used in 59 individuals. Reconstruction was performed using the stomach in 219 patients and colon interposition in 8 patients.

**TABLE 1:**
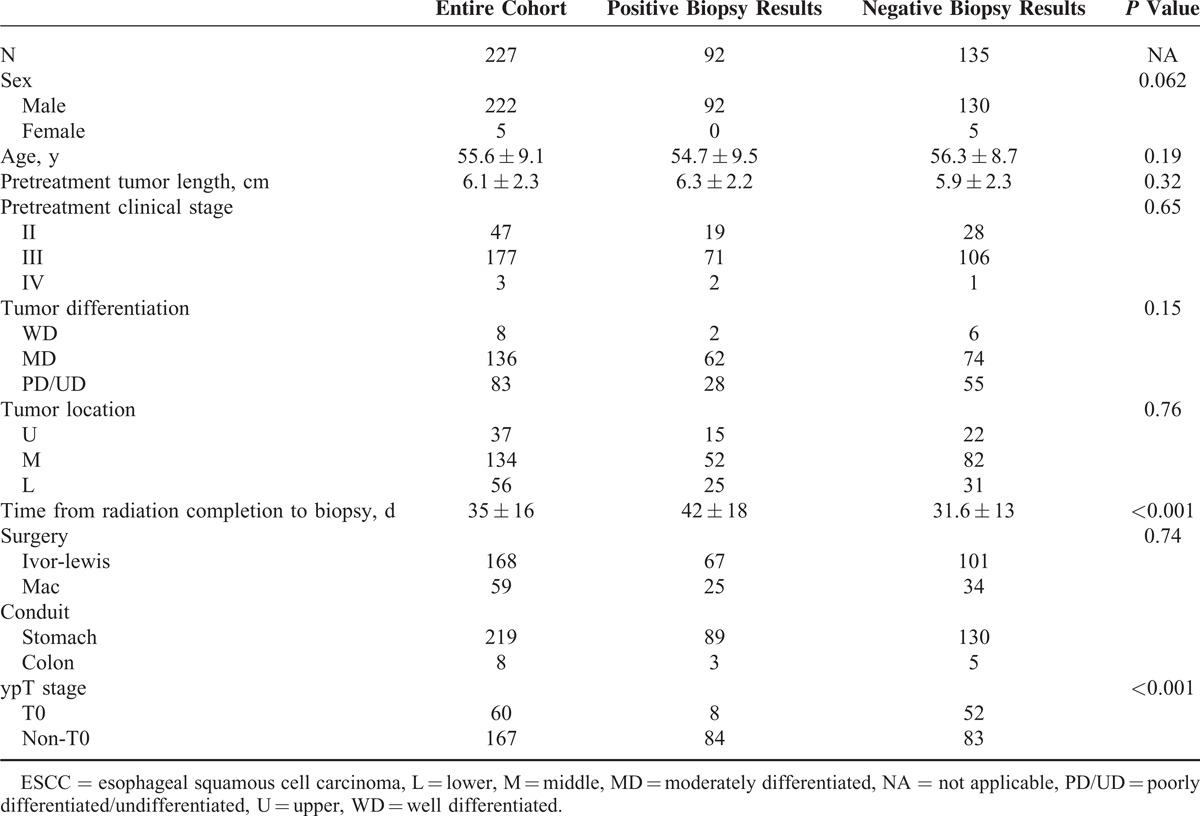
Demographic Characteristics of ESCC Patients (n = 227)

### Correlations Between Findings on Endoscopic Biopsy and Clinical/Pathological Variables

Biopsy results were negative in 135 (59.4%) patients and positive in 92 patients (40.6%; Table 1). Demographic and clinical characteristics did not differ based on this outcome. The mean time between completion of radiation therapy and surgery was significantly longer in patients with negative biopsy results (*P* < 0.001), but pCR was more likely achieved (*P* < 0.001). The sensitivity, specificity, PPV, NPV, and accuracy of endoscopic biopsy following nCRT were 50.2%, 86.7%, 91.3%, 38.5%, and 57.0%, respectively.

### Factors Associated With FN Biopsy Findings

FN results were identified in 61.5% (83/135) of the study patients. Table 2 shows the results of univariate analysis of the variables associated with FN biopsy findings. The type of endoscopic lesion was the only significant factor associated with FN biopsy results, whereas tumor length and age showed a borderline association. The thoroughness with which these biopsies were performed (as reflected by the total number of biopsies) did not show an association with the likelihood of FN results. Multivariable logistic regression analysis showed that the type of endoscopic lesion was the only independent predictor of FN biopsy findings. The odds ratios of pCR in patients with negative biopsy findings were 14 (95% CI, 8–30; *P* = 0.01), 1.82 (95% CI, 0.4–7.9; *P* = 0.43), 6.5 (95% CI, 1.4–30.4; *P* = 0.02), 4.4 (95% CI, 0.94–21; *P* = 0.06), and 1.2 (95% CI, 0.3–5.2; *P* = 0.81) in the normal, ulcer, scar, other findings, and stricture subgroups (compared with the reference tumor subgroup), respectively (see Table 3). The sensitivity, specificity, PPV, and NPV for each type of lesion are summarized in Table 4. The NPV was 77.8%, 61.9%, 52.6%, 30.3%, 23.1%, and 20.0% in the normal, scar, other findings, ulcer, stricture, and tumor categories, respectively (*P* < 0.001).

**TABLE 2:**
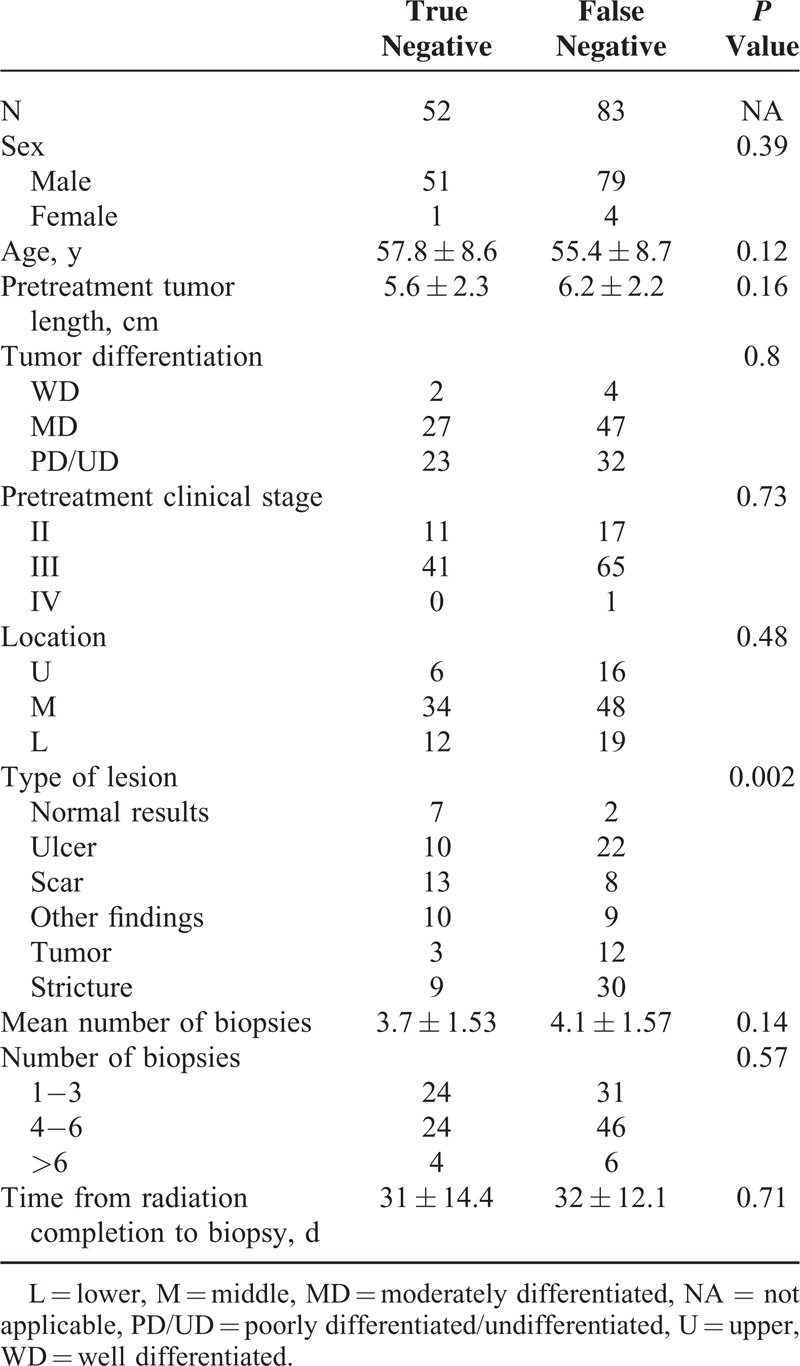
Variables Associated With the Likelihood of Negative Biopsy Findings: Results of Univariate Analysis

**TABLE 3:**
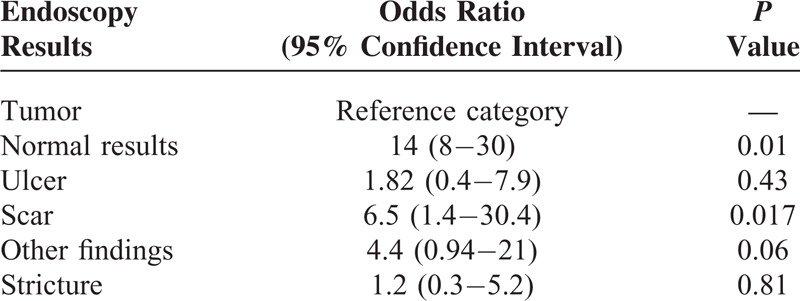
Results of Multivariable Analysis

**TABLE 4:**
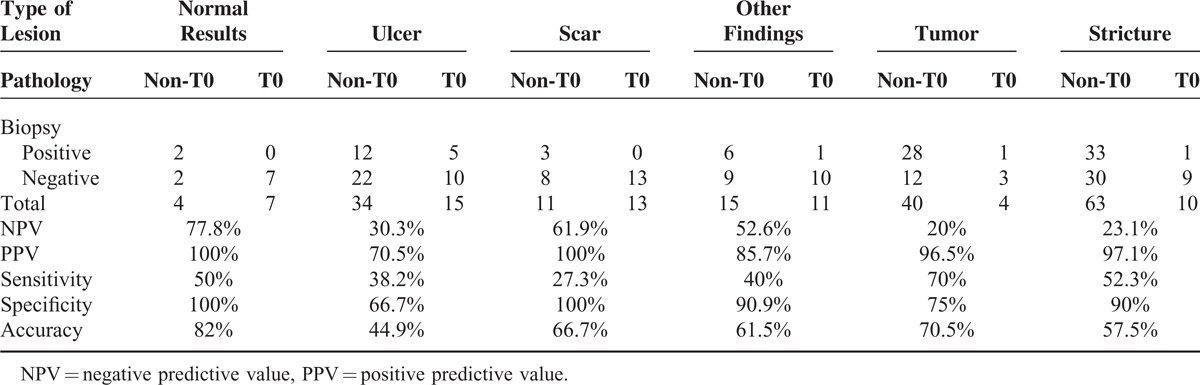
Type of Endoscopic Lesions and Predictive Power of Endoscopic Biopsies

## DISCUSSION

Endoscopic biopsy after nCRT is considered to have high PPV but low NPV for the prediction of the presence of residual cancer in patients with esophageal cancer.^5–8^ Positive biopsy findings after nCRT clearly indicate the presence of a residual tumor (ie, high PPV). However, negative biopsy results do not give sufficient diagnostic confidence to rule out the presence of a residual malignancy (ie, low NPV). Because NPV varies according to the prevalence of the condition under investigation, we hypothesized that the NPV of endoscopic biopsies would be higher in the ESCC group due because they have a greater likelihood of achieving pCR. In our cohort, consisting solely of ESCC patients and characterized by a pCR rate of 26%, we were able to achieve an NPV of 38.5%, which was higher than those reported in previous investigations (Table 5). However, the NPV obtained in our study remains too low to justify a “wait-and-see” policy following nCRT. Indeed, it is expected that up to 61.5% of patients with negative biopsy results will develop local recurrences if not surgically treated. Despite such negative findings, we have successfully identified, for the first time, the most significant factors associated with FN biopsy results. The extent of disagreement between the results of endoscopic biopsies and the findings on postsurgical pathology was found to be significantly associated with the type of residual lesion (ie, stricture, tumor, ulcer, scar, other findings, or normal) detected on endoscopy. We believe that these findings may have important implications in the management of patients following nCRT.

**TABLE 5:**
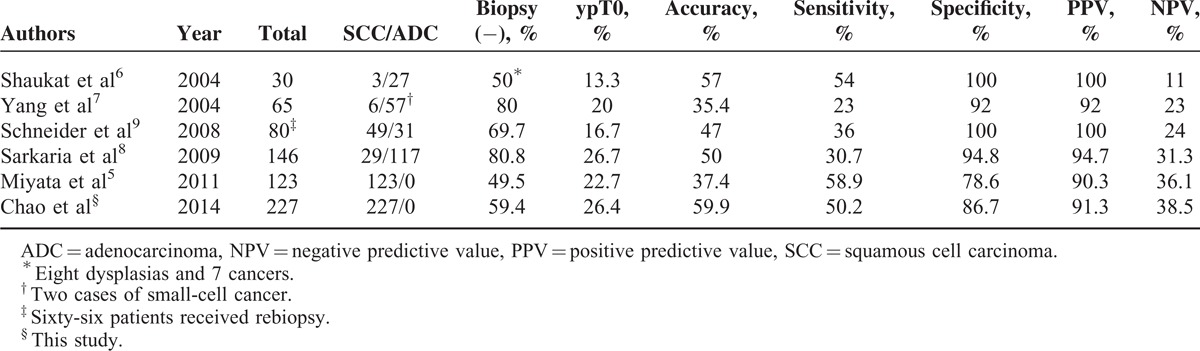
Published Series Comparing the Predictive Power of Endoscopic Biopsies After Chemoradiotherapy

According to the Response Evaluation Criteria in Solid Tumors and World Health Organization criteria, clinical complete response at the primary site is defined as “disappearance of tumor lesion” and the “absence of cancer cells in biopsy specimens.”^18,19^ However, the definition of “disappearance of tumor” is not univocal following nCRT. Even in the absence of a tumor, various types of mucosa lesions may be observed on endoscopy, each of them potentially having a different clinical significance. In our study, we classified nontumoral lesions into 5 distinct categories: normal, ulcer, scar, other findings, and stricture. As shown in Table 3, the NPVs in the ulcer (30.3%) and stricture (23.1%) categories were as poor as that detected for the reference category (ie, the tumor category). Obviously, the presence of cicatricial stenoses of the esophagus following nCRT prevents endoscopic examination of the entire esophagus. In this context, biopsy results should be considered unreliable. The presence of ulcers is not uncommon following nCRT. According to Japanese guidelines, ulcers should be considered as a nonclinical complete response at the primary tumor site following definitive chemoradiotherapy and represent an indication for salvage resection.^20^ Based on our findings, we also suggest that the presence of a nonhealing ulcer following nCRT should be considered a sign of persistent local disease. Meanwhile, the “other findings” category is a heterogeneous mixture and comprises a wide variety of endoscopic findings, including granular protruded lesions, erosion, and lugol-voiding lesions. Despite a slight improvement in NPV (51%), this value was too low to justify a “wait-and-see” policy. Consequently, we believe that endoscopic biopsy after nCRT is not needed, and scheduled resection should not be delayed in patients with lesions classified as ulcer, other findings, or stricture. In contrast, endoscopic biopsy is necessary in patients presenting endoscopic findings classified as either scar or normal following nCRT. Although an NPV of 61.9% to 77.8% is too low to warrant a nonsurgical approach, we believe that an organ-preservation strategy (consisting of close surveillance and postponement of scheduled surgery) represents a reasonable alternative to immediate surgical resection. Notably, this approach is supported by the recent study by Shapiro et al.^21^ By analyzing the distribution of residual cancer after nCRT in a total of 102 patients with esophageal malignancies, the authors identified a strong “layer-dependent” tumor regression pattern. The luminal side (mucosal and submucosal layers) had the highest likelihood of having residual cancer, whereas the highest pCR rate was observed for lesions located in the adventitia. In this scenario, luminal regrowth represented a major recurrence pattern that could be successfully identified through a close endoscopic surveillance before progression to an unresectable stage. Additional studies with larger sample sizes are needed to confirm our hypothesis.

Some caveats of our research merit comment. First, the study has a retrospective nature, and the enrolment period was somewhat long. Endoscopy was performed by expert gastroenterologists, but observer bias cannot be ruled out. Second, the endoscopic findings were divided into 6 categories thus diluting the cohort within each subgroup. It is difficult to have enough statistical power using such small groups; a larger cohort is recommended to be able to make strong conclusions as well as allow for more conclusive statistical analysis. Third, endoscopic findings may be helpful for predicting local pCR but not complete pCR. Finally, the radiation dose used for nCRT was lower than that currently used in routine practice (30 vs 45–50.4 Gy), which may have resulted in different response patterns. Future validation of our findings with larger sample sizes and different nCRT protocols is warranted before making definite recommendations.

## CONCLUSION

In ESCC patients, the FN rate of endoscopic biopsy after nCRT is associated with the type of residual lesion.
